# Retinal thickness as a biomarker of cognitive impairment in manifest Huntington’s disease

**DOI:** 10.1007/s00415-023-11720-3

**Published:** 2023-04-20

**Authors:** Ane Murueta-Goyena, Rocío Del Pino, Marian Acera, Sara Teijeira-Portas, David Romero, Unai Ayala, Tamara Fernández-Valle, Beatriz Tijero, Iñigo Gabilondo, Juan Carlos Gómez Esteban

**Affiliations:** 1 Neurodegenerative Diseases Group, Biocruces Bizkaia Health Research Institute, Barakaldo, Spain; 2 Department of Neurosciences, Faculty of Medicine and Nursery, University of the Basque Country (UPV/EHU), 48930 Leioa, Spain; 3 Biomedical Engineering Department, Faculty of Engineering, Mondragon University, Mondragon, Spain; 4 Neurology Department, Cruces University Hospital, Osakidetza, Barakaldo, Spain; 5 IKERBASQUE, The Basque Foundation for Science, Bilbao, Spain

**Keywords:** Huntington’s disease, Retina, Optical coherence tomography, Cognition

## Abstract

**Background:**

Cognitive decline has been reported in premanifest and manifest Huntington’s disease but reliable biomarkers are lacking. Inner retinal layer thickness seems to be a good biomarker of cognition in other neurodegenerative diseases.

**Objective:**

To explore the relationship between optical coherence tomography-derived metrics and global cognition in Huntington’s Disease.

**Methods:**

Thirty-six patients with Huntington’s disease (16 premanifest and 20 manifest) and 36 controls matched by age, sex, smoking status, and hypertension status underwent macular volumetric and peripapillary optical coherence tomography scans. Disease duration, motor status, global cognition and CAG repeats were recorded in patients. Group differences in imaging parameters and their association with clinical outcomes were analyzed using linear mixed-effect models.

**Results:**

Premanifest and manifest Huntington’s disease patients presented thinner retinal external limiting membrane-Bruch’s membrane complex, and manifest patients had thinner temporal peripapillary retinal nerve fiber layer compared to controls. In manifest Huntington’s disease, macular thickness was significantly associated with MoCA scores, inner nuclear layer showing the largest regression coefficients. This relationship was consistent after adjusting for age, sex, and education and p-value correction with False Discovery Rate. None of the retinal variables were related to Unified Huntington’s Disease Rating Scale score, disease duration, or disease burden. Premanifest patients did not show a significant association between OCT-derived parameters and clinical outcomes in corrected models.

**Conclusions:**

In line with other neurodegenerative diseases, OCT is a potential biomarker of cognitive status in manifest HD. Future prospective studies are needed to evaluate OCT as a potential surrogate marker of cognitive decline in HD.

## Introduction

Huntington’s disease (HD) is a progressive neurodegenerative disorder caused by the expansion of the cytosine-adenine-guanine (CAG) repeat region of the Huntingtin gene (*HTT*). Individuals with 40 CAG repeats or more in *HTT* gene would invariably develop HD over the course of their lifespan [1]. The disease is clinically characterized by alterations of motor function, cognitive impairment and psychiatric symptoms [2], but the diagnosis primarily relies on the presence of motor symptoms. Therefore, subjects with 40 CAG repeats or more that do not meet the clinical criteria for HD are often referred to as premanifest HD. Identifying clinical and imaging prognostic markers in premanifest HD subjects would offer a sensitive time period for early-interventions.

Visual abnormalities are being increasingly acknowledged to be present from early or pre-symptomatic stages in HD. A recent systematic review reported evidence of visual perceptual deficits in HD probably related to alterations in the structural and functional integrity of the afferent visual pathway [3]. In this regard, retinal optical coherence tomography (OCT) is able to measure retinal structure non-invasively and has been explored as a possible neuroimaging technique for finding biomarkers of disease progression in HD, as in other neurodegenerative diseases like multiple sclerosis [4], Parkinson’s disease [5, 6] or Alzheimer’s disease [7]. Thickness reduction of the peripapillary retinal nerve fiber layer has already been demonstrated in HD, regardless of disease stage [8–11]. However, macular metrics have not been studied in depth and the reported findings are insufficient to draw conclusions about their usefulness for disease monitoring [8, 10–14].

Cognitive impairment is one of the cardinal symptoms of HD, and clinical trials are making efforts to include cognition as a clinical endpoint to test the efficacy of neuroprotective agents [15]. Thus, finding sensitive and reliable biomarkers for cognitive outcomes is of keen interest in HD. To our knowledge, there is no study systematically evaluating the use of OCT in HD for revealing potential eye biomarkers of clinical outcomes, including cognition. We aimed to investigate macular and peripapillary changes in HD, evaluate whether there was any association of retinal parameters with disease outcomes, focusing primarily on cognitive outcomes, and test whether these findings were also present in premanifest HD.

## Methods

### Design and participants

A cross-sectional observational study was performed on 36 HD subjects (16 premanifest and 20 manifest) and 36 matched controls. Premanifest and manifest carriers of HD were prospectively recruited from November 2020 to September 2022 in the outpatient Neurology Department at Cruces University Hospital. Age, sex, years of education, age at disease onset, and CAG repeat size in both alleles of the *HTT* gene were recorded. Disease severity was tested with Unified Huntington’s disease Rating Scale (UHDRS) and global cognition with Montreal Cognitive Assessment (MoCA). Subjects with a MoCA score lower than 26 were considered to have Mild Cognitive Impairment (MCI) [16]. HD disease burden score was calculated as ([CAG − 35.5] × age) [1]. Controls were retrospectively recruited at the Department of Ophthalmology of the same hospital, and subjects matched for age, sex, smoking and hypertension status were selected from the OCT registry for the current study.

All HD and control participants completed a comprehensive questionnaire on current comorbidities to check for the following systemic exclusion criteria: diagnosis of any type or grade of diabetes, uncontrolled or resistant elevated blood pressure, history of consumption of drugs or medications known to induce retinal toxicity or cognitive impairment, chronic inflammatory systemic diseases (e.g. lupus erythematosus, sarcoid, Behcet’s disease), history of brain trauma or central nervous system diseases different from HD. Participants with well-controlled hypertension without complications were included in the study.

All participants underwent a complete ophthalmologic examination including pupillary reflexes, refraction, visual acuity, color discrimination, ocular surface examination, and spectral domain OCT. Spherical equivalent refractive error above 4.00 diopters or more than 3.00 diopters of astigmatism or any ocular or systemic pathological condition, except HD, potentially influencing retinal OCT measures were considered exclusion criteria. Participants unable to properly collaborate in OCT images acquisition were not eligible for the study.

The study protocol was approved by the regional Basque Clinical Research Ethics Committee. All participants gave written informed consent prior to their participation in the study, in accordance with the tenets of the Declaration of Helsinki.

### Spectral domain optical coherence tomography (OCT)

Macular retinal thickness was assessed using the Spectralis spectral-domain OCT system (HRA2 Acquisition Module version 6.16.6.0, Heidelberg Engineering, Heidelberg, Germany) with an imaging acquisition protocol that has been described elsewhere [5, 6]. All OCT images fulfilled quality control criteria from OSCAR-IB consensus [17]. Macular volumetric and peripapillary scans were exported in raw format (*.vol) and processed in AURA tools as previously described [18]. The average thickness of the macula (6-mm disc), the thickness in the central region (1-mm diameter disc), parafoveal area (1- to 3-mm diameter ring adjacent to the foveal zone) and the perifoveal area (3- to 6-mm diameter ring adjacent to the parafovea) were computed by averaging the point-by-point thicknesses in each area. The thicknesses were calculated for the whole retina and its layer complexes. The average peripapillary retinal nerve fiber layer (pRNFL) and its temporal sector thickness were calculated. We used the scan focus metric of the macula—registered in the Spectralis OCT- as a surrogate of the refractive error of the eye. Thicknesses of both eyes were averaged unless any pathological condition affected one of the eyes, in which case only measures of the healthy eye were included in the analyses.

### Statistical analysis

Statistical analysis was done in RStudio (version 2022.07.0). Group differences of categorical variables were tested using Chi-square or Fisher’s exact test, as appropriate. Quantitative variables were described using mean and standard deviation. Normal distribution of data was visually inspected. Group comparisons of normally distributed demographic and clinical variables were done with T test and non-normally distributed data assessed with Mann–Whitney U test. Pearson’s correlation coefficient was calculated for testing the association between continuous variables. The analyses of OCT parameters were conducted using linear mixed-effect models (LMM) to account for the inter-eye correlation within participants using *lme4* and *lmerTest* packages. A variable was created to group together premanifest HD with their matched controls and manifest HD with their respective matched controls (*match* variable). Retinal parameters were used as dependent variables and group (HD vs. control), match and their interaction as the fixed effect. A random intercept for the subject was introduced. To test the association between OCT-derived metrics and clinical variables, the retinal parameters were used as predictors in univariable linear regressions (unadjusted models). Then, multivariable linear regressions were fitted to control for confounding factors, including age and sex. Education was also included as a covariate in adjusted models when predicting MoCA scores. Diagnostic plots were created to test for regression model assumptions. False Discovery Rate (FDR) corrected *p* values (pFDR) were calculated to correct for multiple comparisons. *p-values* lower than 0.05 were considered statistically significant.

## Results

### Sample characteristics

We included 16 premanifest HD carriers (32 eyes) and 20 manifest HD patients (38 eyes). Control group consisted of 36 subjects (72 eyes) matched for age, sex, smoking status and hypertension status. One eye from an HD patient was excluded because of amblyopia, and another eye from the same group was excluded because of the presence of an epiretinal membrane.

Demographics and clinical characteristics of participants are listed in Table 1. There were no statistically significant differences between premanifest and manifest HD in the proportion of females (75% and 55%, respectively, *p* = 0.301) or smokers (25% and 42%, respectively, *p* = 0.91) but premanifest subjects were significantly younger (*p* < 0.001).

**Table 1:** Demographics and clinical characteristics

	Pre-manifest HD	Pre-manifest control	HD	HD control	*p* value*
*n*	16	16	20	20	
Age (years old)	46 ± 11	46.1 ± 11.1	53.4 ± 8.2	53.2 ± 8.3	–
Sex, *n* females (%)	12 (75%)	12 (75%)	11 (55%)	11 (55%)	–
Smoker, *n* (%)	8 (25%)	8 (25%)	8 (20%)	8 (20%)	–
HT, *n* (%)	1 (6.3%)	1 (6.3%)	1 (5%)	1 (5%)	–
Scan focus (D)	-0.2 ± 1.7	-0.3 ± 1.5	0.2 ± 1.5	0.5 ± 1.4	–

	Pre-manifest HD		Manifest HD		*p* value
	Mean ± SD	Range	Mean ± SD	Range	
Disease duration (years)	NA	NA	4 ± 2.2	1–7	–
UHDRS	1.75 ± 2.0	0–5	29 ± 16.5	9–68	< 0.0001
CAG repeats, long allele	42.2 ± 1.8	40–45	42.9 ± 2.1	40–49	–
CAG repeats, short allele	17.9 ± 4.2	11–25	18.2 ± 3.3	14–26	–
Disease burden	301.4 ± 95.3	180–501.5	390.8 ± 85.3	288–607.5	0.006
MoCA	25.1 ± 3.2	17–29	20.1 ± 6.1	4–29	0.005
Education (years)	13.4 ± 3.9	6–19	11.9 ± 4.1	0–18	–

*CAG* cytosine-adenine-guanine; *D* diopters; *HD* Huntington's disease; *MoCA* montreal cognitive assessment, *UHDRS* Unified Huntington Disease Rating Scale, *NA* not applicable

*p values were computed for hypothesis testing between pre-manifest and manifest HD

Nine out of 16 (56.3%) premanifest subjects showed motor symptoms with a mean UHDRS score of 1.75 ± 2.0, none of the latter scoring more than 5 on UHDRS. Disease burden score was significantly higher in manifest HD than in premanifest HD (*p* = 0.006). The mean MoCA score was 20.1 ± 6.1 in manifest HD, which was significantly lower than in premanifest HD (25.1 ± 3.2, *p* = 0.005). Nevertheless, 31.3% of premanifest HD showed a MoCA score compatible with MCI. MoCA score was significantly correlated with UHDRS in manifest HD (*r* =  − 0.45, *p* = 0.004), but not in premanifest HD (*r* = – 0.15, *p* = 0.573).

Mean scan focus was similar between manifest HD and controls (*p* = 0.575), premanifest HD and controls (*p* = 0.881), and did not differ between premanifest and manifest HD (*p* = 0.306).

### Group differences in OCT metrics

**Fig. 1:**
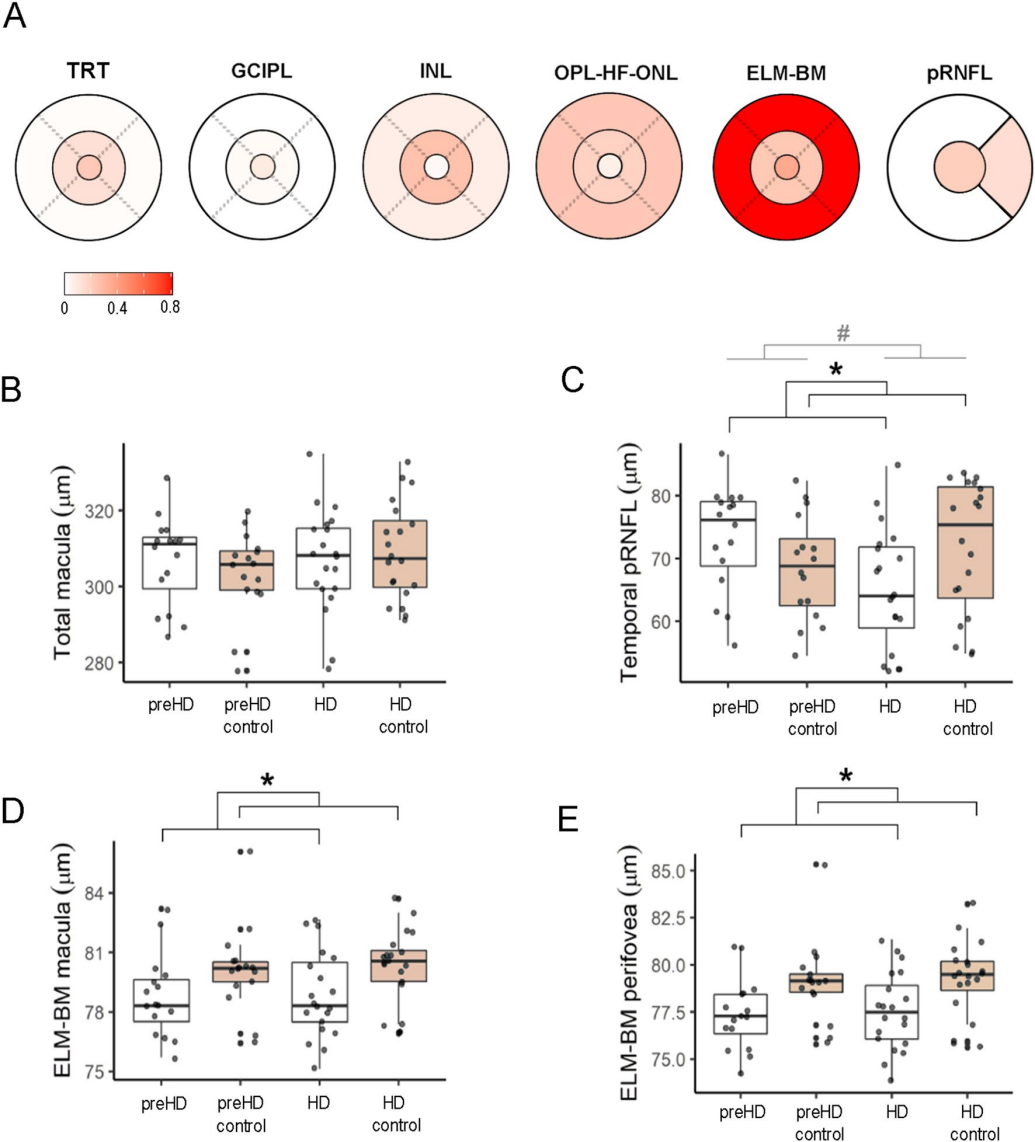
**A** Visual representation of the effect size (Cohen’s d) for thickness differences between HD and controls in each retinal layer and area. In pRNFL, the central circle represents the average pRNFL thickness and the right sector, the temporal pRNFL. Boxplots of **B** Total Macular Thickness in 6-mm diameter ETDRS disc, **C** temporal sector of pRNFL, **D** ELM-BM thickness in 6-mm diameter ETDRS disc, **E** ELM-BM thickness in 3- to 6-mm diameter ring surrounding the parafovea. White boxplots correspond to HD patients and red boxplots to matched controls. The “pre-” prefix indicates premanifest HD and matched controls of these patients.*Significance level of *group* factor (HD vs. control). *p < 0.05, **p < 0.01; #Significance level of *group* x *match* interaction factor. *TRT* total retinal thickness; *GCIPL*, ganglion cell-inner plexiform layer complex; *INL* inner nuclear layer; *OPL-HF-ONL* outer plexiform layer-Henle fiber-outer nuclear layer complex; *ELM-BM* external limiting membrane-bruch’s membrane complex; *pRNFL* peripapillary retinal nerve fiber layer

Total macular thickness was not significantly different between HD patients and matched-controls, nor was it between premanifest and their matched-controls (Table 2). Likewise, there were no significant differences in 3 out of 4 macular layer or layer complex thicknesses between HD and controls. However, we observed that the thickness of ELM-BM complex, particularly in the perifovea, was significantly thinner in premanifest and manifest HD compared to controls (Fig. 1). We also found a non-significant decrease in the average pRNFL thickness in manifest HD compared to matched controls (absolute difference 4.9 µm). However, *group* effect on the temporal pRFNL thickness was significant (*p* = 0.025), and this was mainly driven because temporal pRNFL was 6.8 µm thinner in manifest HD vs. matched controls (group × match, *p* = 0.011).

**Table 2:** Comparison of retinal thickness in pre-manifest and manifest HD vs. matched-controls

	Pre-manifest HD	Pre-manifest control	HD	HD control	Group	Match	Interaction
**Total retinal thickness**							
Macula	306.82 ± 11.75	303.02 ± 10.75	306.92 ± 13.86	309.11 ± 12.69	–	–	–
Perifovea	297.01 ± 11.71	293.95 ± 11.11	297.3 ± 13.74	299.98 ± 12.94	–	–	–
Parafovea	342.73 ± 14.13	336.53 ± 12.05	341.84 ± 15.33	343.16 ± 16.29	–	–	–
Central	223.43 ± 16.29	219.8 ± 12.32	225.6 ± 15.14	222.53 ± 13.43	–	–	–
**GCIPL**							
Macula	71.69 ± 3.82	70.6 ± 4.96	71.34 ± 6.22	72.09 ± 4.95	–	–	–
Perifovea	65.97 ± 4.09	65.07 ± 5.06	65.69 ± 5.83	66.37 ± 5.44	–	–	–
Parafovea	94.92 ± 5.17	92.93 ± 6.15	93.93 ± 8.4	95.25 ± 6.55	–	–	–
Central	31.63 ± 5.21	32.27 ± 5.61	33.77 ± 6.03	32.3 ± 4.83	–	–	–
**INL**							
Macula	33.69 ± 1.88	33.25 ± 1.96	34.19 ± 2.28	33.97 ± 2.3	–	–	–
Perifovea	32.44 ± 2.06	32.31 ± 1.9	32.97 ± 2.13	32.78 ± 2.37	–	–	–
Parafovea	39.75 ± 2.71	38.29 ± 3.03	40.06 ± 3.31	39.69 ± 3.68	–	–	–
Central	14.82 ± 3.64	14.41 ± 3.24	15.91 ± 3.74	16.02 ± 4	–	–	–
**OPL-HF-ONL**							
Macula	89.53 ± 5.25	87.25 ± 6.75	90.73 ± 5.7	89.92 ± 6.71	–	–	–
Perifovea	84.05 ± 5.09	81.59 ± 6.73	85.44 ± 5.96	84.67 ± 6.62	–	–	–
Parafovea	103.72 ± 6.49	101.97 ± 8.1	104.61 ± 5.68	103.51 ± 8	–	–	–
Central	97.33 ± 8.34	96.09 ± 7.86	96.4 ± 6.25	96.51 ± 6.98	–	–	–
**ELM-BM**							
Macula	78.76 ± 2.07	79.97 ± 1.91	78.89 ± 2.2	80.27 ± 1.95	0.036	–	–
Perifovea	77.38 ± 2.01	79.12 ± 2.0	77.55 ± 2.13	79.24 ± 2.04	0.010	–	–
Parafovea	81.65 ± 2.76	82.15 ± 2.45	81.65 ± 2.84	82.31 ± 2.22	–	–	–
Central	73.14 ± 4.24	71.37 ± 3	73.01 ± 4.36	71.92 ± 3.68	–	–	–
**pRNFL**							
Average	99.43 ± 7.84	97.28 ± 9.24	96.97 ± 7.76	101.83 ± 9.33	–	–	–
Temporal sector	73.24 ± 9.84	68.14 ± 8.52	65.04 ± 9.94	71.79 ± 11	0.025	–	0.011

Control participants were matched by age, sex, smoking status and hypertension status with HD patients. *p* values were calculated using linear mixed-effects models. All measurements are in micrometers. Group factor indicates whether participants are HD or controls, whereas Match is a two-level factor for grouping pre-manifest HD and their matched-controls and manifest HD with their matched controls

*GCIPL*x ganglion cell-inner plexiform layer; *ELM-BM* retinal complex including external limiting membrane, inner and outer segments of photoreceptor, and retinal pigment epithelium; *INL* inner retinal layer; HD, Huntington’s disease; *OPL-HF-ONL* outer plexiform-Henle fiber-outer nuclear layer complex; *pRNFL* peripapillary retinal nerve fiber layer; *SD* standard deviation

### Association between retinal parameters and clinical outcomes

We next explored whether retinal parameters were associated with scores of clinical variables, including disease duration, UHDRS, global cognition, CAG repeats (both alleles) and disease burden. We performed separate analyses for premanifest and manifest HD.

The results showed that, in manifest HD, the macular thickness and all of its layers except ELM-BM were significantly associated with global cognition (Table 3), explaining 28% up to 55.4% of MoCA variability. INL showed the highest regression coefficient (unadjusted *β* = 1.84, pFDR = 0.002), whereas Total Retinal Thickness explained the highest proportion of MoCA variability (55.4%). Age, sex and education-adjusted models confirmed these results.

**Table 3:** Linear regression models for cognitive and motor scores in HD

	Unadjusted			Adjusted		
	*β*	SE	*p* value	*β*	SE	*p* value
**MoCA score**						
Total retinal thickness	0.34	0.07	0.002**	0.31	0.07	0.007**
GCIPL	0.63	0.19	0.009**	0.43	0.21	0.088
INL	1.84	0.46	0.002**	1.51	0.39	0.005**
OPL-ONL	0.75	0.18	0.002**	0.64	0.15	0.002**
ELM-BM	− 0.28	0.68	0.76	0	0.67	0.998
pRNFL	0.49	0.18	0.022*	0.38	0.18	0.087
pRNFL, temporal	0.18	0.15	0.351	0.11	0.13	0.544
**UHDRS score**						
Total retinal thickness	− 0.46	0.26	0.326	− 0.70	0.28	0.262
GCIPL	− 1.06	0.58	0.339	− 1.38	0.61	0.110
INL	− 2.85	1.50	0.750	− 3.38	1.56	0.114
OPL-ONL	− 1.02	0.63	0.231	− 1.32	0.68	0.125
ELM-BM	2.34	1.72	0.272	2.26	2.03	0.354
pRNFL	− 0.58	0.54	0.358	− 1.05	0.62	0.171
pRNFL, temporal	− 0.14	0.40	0.722	− 0.13	0.43	0.758

*p* values were corrected with False Discovery Rate (FDR) for multiple comparisons. In adjusted models age, sex, and education were introduced as covariates for predicting MoCA score, whereas age and sex was used in UHDRS models. Significant p-values are highlighted with asterisks (*p < 0.05, **p < 0.01)

*GCIPL* ganglion cell-inner plexiform layer; *ELM-BM* retinal complex including external limiting membrane, inner and outer segments of photoreceptor, and retinal pigment epithelium; *INL* inner retinal layer; *MoCA* montreal cognitive assessment; *OPL-ONL* outer plexiform-outer nuclear layer complex; *pRNFL* peripapillary retinal nerve fiber layer; *UHDRS* Unified Huntington’s disease Rating Scale

The average pRNFL thickness was also significantly associated with the MoCA score in the unadjusted model (*β* = 0.48, pFDR = 0.02) but not after controlling for the effect of age, sex, and education (*β* = 0.038, pFDR = 0.09). Similarly, the thickness of the Total Retina, GCIPL, INL and perifoveal OPL-ONL complexes were significantly but marginally associated with UHDRS scores in the adjusted model. However, they lost significance with FDR *p* value correction.

Analyzing different sectors of the macular or temporal pRNFL did not result in improved predictions. None of the OCT metrics showed significant pFDR-corrected associations with disease duration, GAC repeats, or disease burden score.

In premanifest HD, unadjusted models showed that GCIPL thickness in the parafoveal area was associated with CAG repeats in the long allele (*β* = 0.18, SE = 0.08, *p* = 0.04), central thickness of INL with disease burden (*β* = 16.4, SE = 5.5, *p* = 0.01), and temporal pRNFL thickness with UHDRS (*β* =  − 0.12, SE = 0.05 *p* = 0.038), but these associations became non-significant after controlling for confounding variables and p-value FDR correction.

## Discussion

In this study, we described the differences in macular layer and pRNFL thicknesses in premanifest and manifest HD compared to age-, sex-, smoking status- and hypertension status-matched controls, and explored whether retinal parameters could be used as biomarkers in HD. As far as we know, this is the first study in the literature that has studied the relationship between retinal parameters and cognition in premanifest and manifest HD. Our study showed that, compared to controls and regardless of disease stage, HD patients had a significant thinning of the macular ELM-BM complex, the outermost layers of the retina. Moreover, compared to controls, temporal pRFNL was significantly thinner in manifest HD but not in premanifest HD, which suggests that atrophy of pRNFL only occurs with advancing clinical stages of HD. In addition, our results evidenced that retinal OCT metrics were significantly associated with cognitive outcomes in manifest HD. Macular INL thickness showed the highest association coefficient with MoCA scores, rendering this retinal layer as a possible candidate biomarker for monitoring cognition in HD. In premanifest HD, none of the retinal parameters was associated with clinical outcomes after multiple comparison corrections of p-values.

Retinal thickness has been measured in HD using OCT in previous cross-sectional studies. As far as we know, 7 case–control studies have been published, from which 4 have included manifest HD, 2 premanifest HD, and 1 both manifest and premanifest HD subjects. From these, 5 studies have explored RNFL changes, and 5 studies the differences in total macular parameters [8, 11–14], while only 1 explored the macula layer by layer [10]. Previous studies are consistent in that the average pRNFL is thinner in HD [11, 14], at least in the temporal sector [8–10], whereas one study failed to find such a difference [12]. This is in line with current results, in which we observed a thinning of the temporal pRFNL. However, this was only present in manifest HD and not premanifest HD. Regarding macular layers, Gulmez Sevim et al. [10] reported a significant thinning of all macular layers in HD compared to controls, except for photoreceptor and retinal pigment epithelium layers. This finding is in contrast to current results, and the discrepancy could be related to differences in eye length or refractive error between HD and controls. In our study, we corroborated that the eye defocus was similar between HD and controls, but Gulmez Sevim et al. [10] did not specify it, being a possible confounder of their conclusions.

In our study, no significant differences in OCT variables were found across premanifest and manifest HD compared to their matched controls with the exception of the ELM-BM complex. The ELM-BM thickness was indeed thinner in HD groups. The ELM-BM mainly contains inner and outer segments of photoreceptors and retinal pigment epithelium. Intriguingly, preclinical studies have shown that, although mutant huntingtin protein is expressed in all nuclear layers of the retina, severe and specific damage of outer segments of photoreceptors is observed in R6 mice [14, 19–21]. Moreover, this photoreceptor degeneration is linked to visual dysfunction and has been reported to be progressive [14, 19]. As far as we know, our study is the first one to describe a thinning of outermost retinal layers in HD in vivo that coincides with the retinal phenotype of HD animal models. The pathologic mechanism behind why ELM-BM thickness decreased in HD patients regardless of the motor condition is beyond the scope of this study and requires further investigation. It might be related to the fact that the membranes of photoreceptors are sensitive to lipid peroxidation [22], a process that has been linked to mutant huntingtin inclusions [23]. Because the amount of ELM-BM decrease was clinically marginal, we assume the structural alterations are subtle, probably linked to shortened segments, slowed turnover of disc shedding in photoreceptors, or a mild atrophy of RPE.

Regarding the clinical utility of OCT measurements as a biomarker of disease progression in HD, we observed that macular thickness metrics were significantly associated with cognitive performance, being the INL thickness the strongest associated factor. This association was only found in manifest HD. The first OCT studies in HD published in the literature found that macular thickness correlated with UHDRS and temporal pRNFL thickness with disease duration. Recently, Amini et al. [11] have also reported that total macular thickness was correlated with disease duration. Gulmez Sevim et al.[10] found that macular GCIPL thickness was significantly correlated with disease duration, disease burden, CAG repeats, UHDRS and patient independence in manifest HD, but Schmid et al. [13] failed to find any association of GCIPL with UHDRS motor and cognitive scores in premanifest HD. In our study, we observed that several retinal metrics were associated with UHDRS or disease duration in both premanifest and manifest HD, but due to marginally significant *p* values, these associations became non-significant after FDR correction.

Contrarily, the association between retinal parameters and cognition was consistent, as it continued to be significant after controlling for the effect of age, sex, education and applying FDR correction to p-values. Studies in other movement disorders, like Parkinson’s disease, have also proposed that retinal changes might represent cognitive impairment more faithfully than other aspects of disease progression [5]. It is worth mentioning that none of the retinal metrics associated with cognition were significantly different from controls or between premanifest and manifest HD. It should be noted that the range of normality in retinal thickness considerably varies among individuals because behavioral [24, 25], genetic [26] and health conditions [27, 28] directly influence retinal layer thickness. However, our findings support the notion that OCT might be useful as a biomarker of cognitive performance in HD. It is now widely reported that mild cognitive impairment might be present prior to HD motor diagnosis. Nearly 40% of premanifest HD meet the criteria for MCI [29], while the prevalence of MCI in manifest HD might be up to 90% [30]. Therefore, cognition seems to be affected early over the course of HD, and retinal OCT might be used for monitoring cognitive deterioration from early stages, but prospective long-term studies with larger sample sizes are required to confirm this hypothesis.

This study has several advantages over previous studies. First, we included both premanifest and manifest HD and compared them with controls matched by age, sex, smoking status and hypertension status, thereby controlling for important demographic, health and lifestyle conditions influencing retinal thickness. Second, this is the first OCT study including cognitive measurement as a clinical outcome in HD. Although cognitive impairment has not been widely accepted as a disease stage indicator in HD literature, there is increasing interest in using cognition as a clinical endpoint. Third, unlike in previous studies, p-values were corrected for multiple comparisons in regression analyses, and as a result, the chances of reporting false positive results have been minimized.

One of the main limitations of the present study is its cross-sectional design, which limits the interpretability of our results in terms of disease monitoring. In addition, our study was limited by a small sample size. Chorea is a major determinant for OCT eligibility, which hampers more advanced HD patients from being included in OCT studies and also pose considerable bias regarding the generalizability of the obtained results to the whole spectrum of HD. Also, a small sample size in combination with p-value adjustments might have prevented us from revealing some important relationships between the retina and clinical outcomes.

In conclusion, our macular and peripapillary OCT findings suggest that retinal thickness might be marginally affected in HD and probably restricted to the most external layers and to the temporal sector of pRNFL. However, INL thickness was significantly and consistently associated with cognitive performance in manifest HD, revealing this OCT-derived parameter as a potential and reliable biomarker for detecting cognitive impairment in more advanced stages of HD. The inclusion of a large sample of premanifest and manifest HD along with matched controls in future studies and their prospective follow-up can help to corroborate the current findings.

## Data Availability

Data available on reasonable request due to ethical restrictions.
